# Enhanced Energy Storage Properties of the Relaxor and Antiferroelectric Crossover Ceramic Enabled by a High Entropy Design

**DOI:** 10.3390/ma18091937

**Published:** 2025-04-24

**Authors:** Yinghao Li, Wei Xiong, Xuefan Zhou, Hang Luo, Ru Guo, Dou Zhang

**Affiliations:** 1 Powder Metallurgy Research Institute, State Key Laboratory of Powder Metallurgy, Central South University, Changsha 410083, China; yinghaoli@csu.edu.cn (Y.L.); zhouxuefan@csu.edu.cn (X.Z.); hangluo@csu.edu.cn (H.L.); dzhang@csu.edu.cn (D.Z.); 2 Light Alloy Research Institute, Central South University, Changsha 410083, China; 3 State Key Laboratory of Precision Manufacturing for Extreme Service Performance, Central South University, Changsha 410083, China; 4 Department of Mechanical and Automation Engineering, The Chinese University of Hong Kong, Shatin, Hong Kong, China

**Keywords:** high entropy ceramic, relaxor ferroelectric–antiferroelectric crossover, polar nanoregions, energy storage properties

## Abstract

In this work, we introduce a high entropy effect in designing a relaxor ferroelectric (RFE)–antiferroelectric (AFE) crossover ceramic by incorporating a high entropy relaxor-like oxide (Pb_0.25_Ba_0.25_Sr_0.25_Ca_0.25_)TiO_3_ with antiferroelectric NaNbO_3_. The results show that the relaxor ferroelectricity of the system is enhanced with increasing NaNbO_3_, and when the new composition reaches the highest configurational entropy, stable energy storage properties can be achieved. This is enabled by a high breakdown strength due to the small grain size and stable slim ferroelectric hysteresis loop with high efficiency due to entropy-stabilized short-range ordered polar nanoregions (PNRs). These findings showcase the potential of this strategy for exploiting new compositions of high-performance electrostatic capacitors.

## 1. Introduction

Ferroelectric materials (FE) have long been an interesting research topic for their potential use in electrostatic capacitors [1,2,3]. There are special members out of this family that show even more intriguing properties that deviate from normal ferroelectric behaviors: one is antiferroelectric (AFE), featuring a double polarization-electric field (P-E) hysteresis loop that results in high energy density but low efficiency ( η ) [4,5]; the other is relaxor ferroelectric (RFE), with a slim hysteresis loop, which offers high efficiency but low breakdown strength (E_B_) [6,7]. Naturally, it is tempting to develop RFE–AFE crossovers to see if they inherit the merits of both members. Many efforts have been made to achieve this goal, mostly via doping and defects engineering [8,9,10,11]. Recently, many turn to NaNbO_3_ (NN) for its relatively large bandgap E (∼3.58 eV), low volatility, and readily tunable AFE–FE transitions that are rich to explore [12,13,14]. For example, Zuo et al. [15] incorporated BaZrO_3_ into NN, which effectively introduces relaxor behaviors and ultimately enhances the energy density of the ceramic; Li et al. [16] introduced Ca_0.7_Sm_0.2_TiO_3_ into NN and achieved a stable relaxor ferroelectric state, which improves the E_B_ and efficiency of the ceramic; Xie et al. [17] investigated the effect of adding BaTiO_3_ (BT) in the NN-BiFeO_3_, and this ternary system shows strong relaxor behaviors at the optimal composition and has its energy density and efficiency simultaneously increased. Moreover, NN has been found to improve the temperature stability of the ceramics [18,19], which is essential for actual applications.

The above studies showcase the benefits of combining RFE with AFE members. It can be seen that a major principle of these strategies is introducing local inhomogeneities, which break down the long-range ordered ferroelectric domains and form short-range ordered substructures known as “nano polar regions” (PNRs) [20,21,22]. Along this line, adding more dopants with distinct physical and chemical properties (e.g., ionic size, valence, etc.) would theoretically facilitate such a phenomenon. However, in practice, the solubility limit of elements oftentimes leads to a secondary phase and poor properties when too many different members are added [23,24]. This is where the recently proposed concept, “high entropy design”, could fit in and make a difference. High entropy design describes an unconventional material design method where the configurational entropy (*S_config_*) of the system is maximized by mixing multiple elements with total- or near-equimolar stoichiometry, and thus, phase purity and stability can be ensured [25,26,27,28]. Recent studies [29,30,31,32,33,34,35] found that relaxor behavior and energy storage performance could be significantly enhanced when applying this method. However, its effectiveness on making high-performance RFE–AFE ceramic has not been fully investigated; especially, the E_B_ of the ceramic needs further improvement.

Here, we develop a new RFE–AFE crossover ferroelectric by combining a high entropy relaxor-like (Pb_0.25_Ba_0.25_Sr_0.25_Ca_0.25_)TiO_3_ (PBSC), which has a high dielectric constant and low loss [36], with an AFE end member NaNbO_3_, which has a high breakdown strength. We demonstrate that, at the optimal composition with the highest *S_config_*, the ceramic has improved E_B_, ultra-high η , and moderate recoverable energy density (*W_rec_*), making it a promising candidate for high-performance energy storage capacitors.

## 2. Materials and Methods

### 2.1. Solid-State Synthesis

The (1-x)(Pb_0.25_Ba_0.25_Sr_0.25_Ca_0.25_)TiO_3_-xNaNbO_3_ (PBSC–NN, x = 0.1, 0.2, and 0.3) powders were prepared by mixing stoichiometric amounts of PbO (99.0%, Sinopharm, *Beijing*, China), BaCO_3_ (99%, Sinopharm), SrCO_3_ (99.0%, Sinopharm), CaCO_3_ (99.0%, Sinopharm), TiO_2_ (98.0%, Sinopharm), Na_2_CO_3_ (99.8%, Sinopharm), and Nb_2_O_5_ (99.99%, Sinopharm). The mixture was wet milled for 12 h and calcined in an alumina crucible at 850 °C for 6 h. The dried powder was uniaxially pressed at 225 MPa to produce 10 mm diameter pellets. These ceramic pellets were sintered at 1250 °C in air for 3 h and then cooled down along with the furnace.

### 2.2. Characterization

The crystal structure of the ceramic was measured by the X-ray diffraction method (λ = 1.5418 Å, D/Max 2250, Rigaku Corporation, Tokyo, Japan). The XRD pattern was fitted by the Rietveld method using the GSASⅡ software (SVN version 5305). The Raman spectra of the ceramic were measured by a DXR3 Thermo Scientific spectrometer (Thermo Scientific, Waltham, MA, USA). The observation of grain morphology and elements analysis were conducted using the scanning electron microscopy (SEM, MIRA4 TESCAN, Brno, Czech Republic) equipped with an energy dispersive spectroscope (EDS, NordlysMax2, Oxford Instruments, Oxford, UK). The grain size distribution of the sintered samples was determined using Image J software (win 64), measuring 200 random-selected grains from the SEM images. The ceramic was coated with gold electrodes for the current-electric field (I-E)/polarization-electric field (P-E) hysteresis loop measurements using a ferroelectric tester (TF Analyzer 2000, aixACCT, Aachen, Germany). The microscopic ferroelectric/piezoelectric properties of the ceramic were analyzed by piezoelectric force microscopy (PFM, Bruker Dimension Icon, Billerica, MA, USA).

## 3. Results and Discussion

### 3.1. Phase Composition and Microstructure

The configurational entropy in perovskite ceramics can be calculated using the following formula [31]:

(1) $$S_{config}=-R[{(\sum_{a=1}^{n}x_{a}lnx_{a})}_{A-site}+{(\sum_{b=1}^{n}x_{b}lnx_{b})}_{B-site}+3{(\sum_{c=1}^{n}x_{c}lnx_{c})}_{O-site}]$$

In the formula, *R* represents the gas constant (*R* = 8.314 J/mol⋅K), and *x_a_*, *x_b_*, and *x_c_* are the mole fractions of the ions at the *A-site*, *B-site*, and *O-site*, respectively [37]. The *S_config_* of (1-x)PBSC–xNN, (x = 0.1, 0.2, and 0.3) are 1.90R, 2.11R, and 2.19R, respectively.

**Figure 1:**
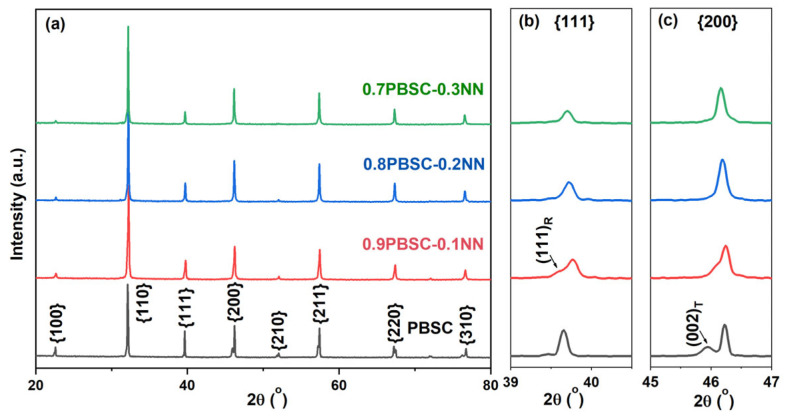
(**a**) XRD patterns of PBSC–NN compositions and the enlargements of (**b**) (111) and (**c**) (200) peaks.

Figure 1 shows the XRD pattern of the (1-x)PBSC–xNN (x = 0.1, 0.2, and 0.3) sintered ceramic. The material is single-phase for all compositions, demonstrating that Na^+^ and Nb^5+^ successfully dissolve into PBSC, and a homogeneous solid solution is formed. The composition dependent (111) and (200) peaks are shown in Figure 1b,c. It can be seen that the PBSC ceramic shows a tetragonal phase structure, as evidenced by the doublet (002)/(200) diffraction peaks and single (111) peak. With the introduction of 0.1NN, (002)/(200) peaks are found to merge together, while there is a (111) peak splitting, indicating the formation of a rhombohedral phase. In general, the stability of perovskites is evaluated by the tolerance factor t [38]:

(2) $$t=\frac{R_{A}+R_{O}}{\sqrt{2}\left(R_{B}+R_{O}\right)}$$

where *R_A_*, *R_B_*, and *R_O_* are the ionic radii of the *A*, *B*, and *O* ions. When 0.9 < *t* < 1, there is a high probability to form a stable cubic perovskite structure; *t* > 1 favors a tetragonal or hexagonal structure; 0.71 < *t* < 0.9 usually leads to the oxygen octahedra tilt and the rhombohedral or orthorhombic structure [39]. Compared with PBSC (t = 1.013), the NN modifier owns a low t of 0.967, inducing the structure transformation. With the further addition of NN, both (111) and (200) peaks do not display any splitting, indicating the formation of a pseudo-cubic phase [40]. The XRD results show that the addition of NN into PBSC can induce the phase transition, enhance the crystal symmetry, and disrupt the ferroelectric ordering. In addition, compared with the PBSC counterpart, the (111) and (200) peaks shift to lower angles with increasing NN content, which indicates that the unit cell is expanded after introducing NN [41].

**Figure 2:**
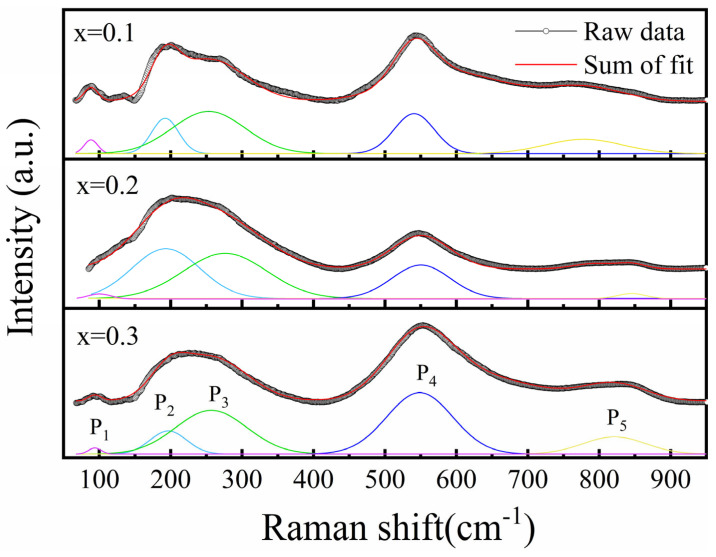
Raman spectra of (1-x)PBSC–xNN (x = 0.1, 0.2, and 0.3) and fitted peaks (P_1_~P_5_).

Raman spectroscopy of the ceramic is performed (Figure 2) to help understand how the local bonding environment evolves as the NN content increases. Five apparent peaks (P_1_~P_5_) are identified by deconvolution of the measured data using the Gaussian function, with each peak corresponding to a vibrational mode of chemical structures. The peak below 150 cm^−1^ (P_1_) is related to the vibration of A-site cations, the middle wavenumber range within 150–400 cm^−1^ can be attributed to the vibration mode of B–O bonds, and the wavenumber region of 400–700 cm^−1^ represents the BO_6_ octahedral vibrations [42]. The intensity and sharpness are significantly reduced at x = 0.2 composition, indicating higher structural disorder and, thus, enhanced relaxor ferroelectricity [15,16,43,44]. Moreover, splitting of the peak at 200~300 cm^−1^ (P_2_, P_3_) represents a coexistence of multiple phases with different crystal structures [45,46], which is in accordance with XRD observations.

**Figure 3:**
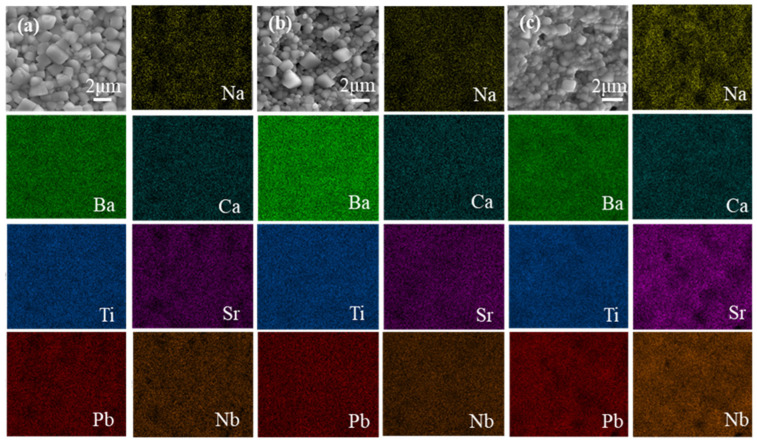
SEM micrographs and EDS elemental mappings of the ceramics: (**a**) 0.9PBSC–0.1NN, (**b**) 0.8PBSC–0.2NN, and (**c**) 0.7PBSC–0.3NN.

Figure 3 shows the surface of the sintered PBSC–NN ceramics and their corresponding EDS results; all elements are homogeneously distributed, and no apparent secondary phase is identified. The average grain sizes of all samples are below 1.5 μm , and that of the PBSC–0.2NN sample is the smallest (1.3 ± 0.7 μm ). A small grain size contributes to the high E_B_ due to increased grain boundaries, which have higher electrical resistivity.

### 3.2. Dielectric Properties

**Figure 4:**
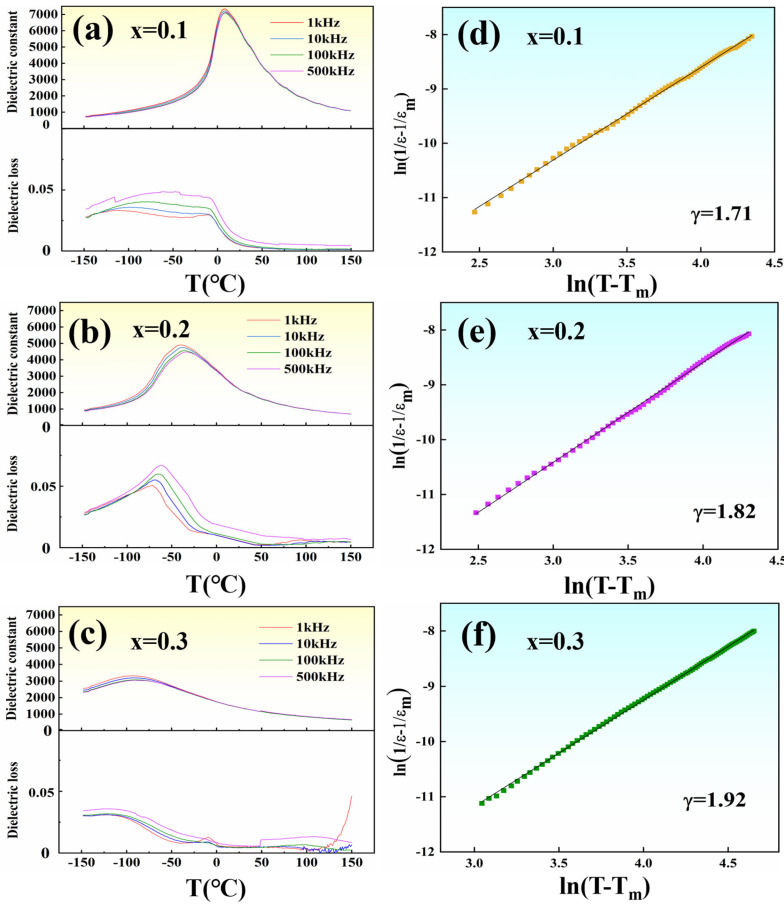
(**a**–**c**) Dielectric constant and loss of (1-x)PBSC–xNN and (**d**–**f**) the corresponding diffusivity of the dielectric constant as fitted by the modified Curie–Weiss law.

The temperature-dependent dielectric constant and loss of the PBSC–NN ceramics are demonstrated in Figure 4. The dielectric constant ( $\varepsilon_{r}$ ) decreases continuously with increasing NN content, the maximum dielectric temperature (*T_m_*) moves towards lower temperature regions, and the reduced $\varepsilon_{r}$ and *T_m_* are linked with disruption of long-range ordered (LRO) ferroelectric domains as a direct result of introducing NN content, which is accompanied by an increase in the configurational entropy (*S_config_*). This is indicative of enhanced relaxor ferroelectricity and is consistent with prior works [47]. Moreover, an apparent frequency dispersion of dielectric permittivity and broader and more diffused *T_m_* peaks were observed as the NN content increased, the latter of which is quantified by the diffusivity value (*γ*), as given by the modified Curie–Weiss law:

(3) $$\frac{1}{\varepsilon_{r}}-\frac{1}{\varepsilon_{m}}=\frac{{\left(T-T_{m}\right)}^{\gamma }}{C}$$

where $\varepsilon_{m}$ and C are the maximum dielectric permittivity and Curie Constant, respectively. Fitted *γ* values of different compositions are shown in Figure 4d–f; a value approaching 2 suggests a deviation from normal ferroelectric behavior and enhanced relaxor ferroelectricity with increasing NN content.

### 3.3. Ferroelectric Properties

**Figure 5:**
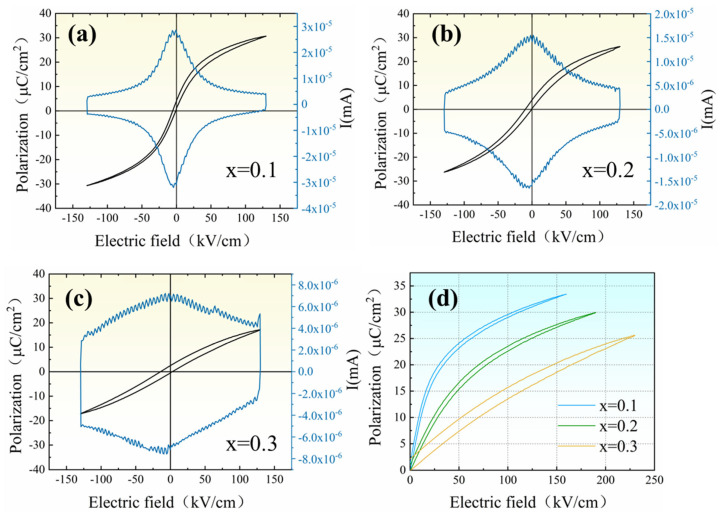
(**a**–**c**) Bipolar P-E and I-E loops and (**d**) the unipolar P-E loops of (1-x)PBSC–xNN ceramics.

Figure 5 demonstrates the ferroelectric P-E and I-E loops of PBSC–NN ceramics; all compositions observe apparent ferroelectric domain switching and a slim P-E hysteresis. We note that the test temperature (RT) is above the *T_m_*, which further confirms the relaxor ferroelectricity of the ceramics. The I-E loop shows a characteristic near-zero-field current peak that becomes more diffused as the NN content increases, which is contributed by the PNRs/paraelectric matrix and is indicative of enhanced relaxor ferroelectricity, all consistent with previous dielectric measurement results (Figure 4). Unipolar P-E loops (Figure 5d) are measured to compare the energy storage properties of different compositions. It is found that the *W_rec_* is improved with increasing NN, reaching 2.4 J/cm^3^ at 0.3NN, which has the highest *S_config_*, whereas the η is maximized at 0.2NN (91%). This is consistent with previous studies [13,16,48], where a solid solution with a high NN content greatly enhances the energy density but suffers from reduced efficiency, which can be understood considering the loss of AFE–FE transitions in pure NN. Here, with the aid of high entropy RFE member PBSC, high efficiency is conserved below 0.2NN content. It is proposed that the high entropy effect not only stabilizes the single-phase solid solution but also facilitates local disorder [49,50,51], including lattice distortion (i.e., size disorder) and charge disorder; such disorder is known to cause random electric fields and spawns PNRs, which contribute to the high efficiency of the PBSC–NN ceramics.

**Figure 6:**
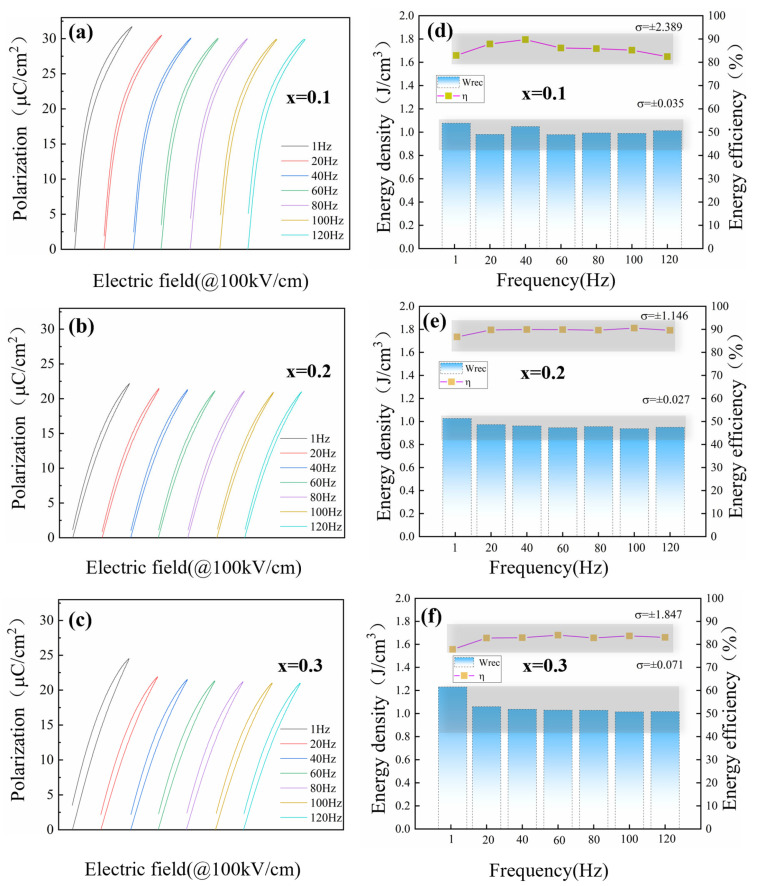
(**a**–**c**) Frequency-dependent unipolar P-E loops and (**d**–**f**) the variation in recoverable energy density (*W_rec_*) and efficiency (η) of (1-x)PBSC–xNN ceramics from 1 to 120 Hz.

**Figure 7:**
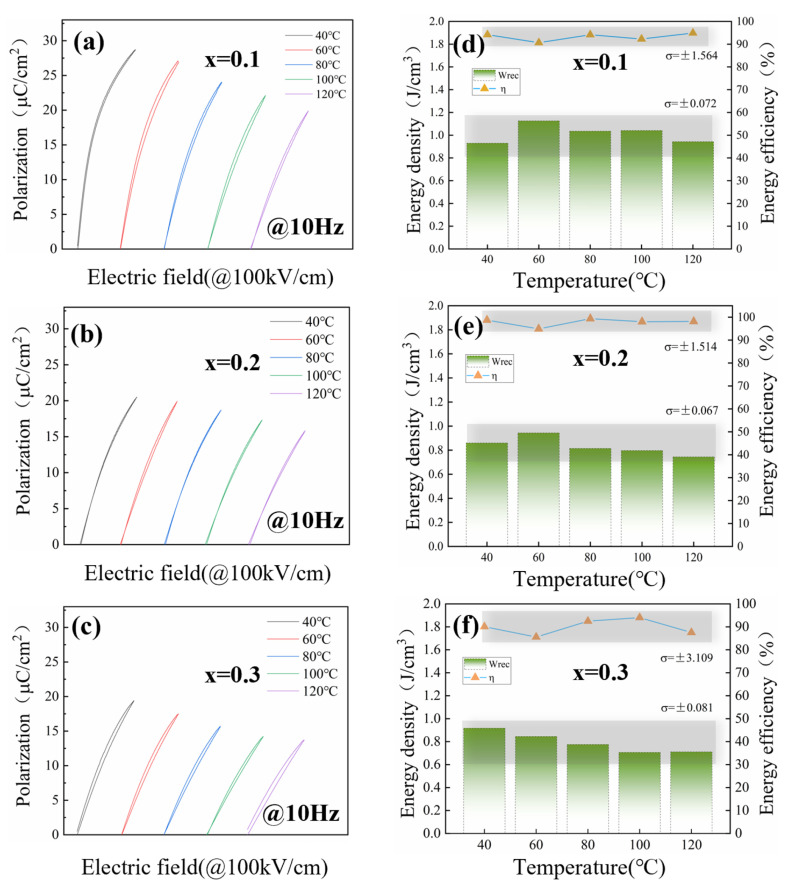
(**a**–**c**) Temperature-dependent unipolar P-E loops and (**d**–**f**) the variation in recoverable energy density (*W_rec_*) and efficiency (η) of the (1-x)PBSC–xNN ceramics from 40 to 120 °C under 10 Hz.

Figure 6 and Figure 7 show the frequency- and temperature-dependent unipolar P-E loops and energy storage properties of PBSC-NN. The variation in *W_rec_* and η can be characterized by the standard deviation (σ) of these values measured from samples in elevating temperatures and frequencies. It is noted that the shape of ferroelectric loops remains slim with elevating temperature, even above the T_m_ temperature, confirming the stable relaxor ferroelectricity of the material. Furthermore, in both tested frequency (1~120 Hz) and temperature ranges (40~120 °C, 10 Hz), the high entropy composition 0.8PBSC–0.2NN demonstrates the most stable performance, as evidenced by the smallest fluctuation of *W_rec_* and η (Figure 6e and Figure 7e). This could be contributed by the entropy-stabilized phase structures and PNRs, which make the material a promising candidate for practical applications of electrostatic capacitors.

### 3.4. PFM

**Figure 8:**
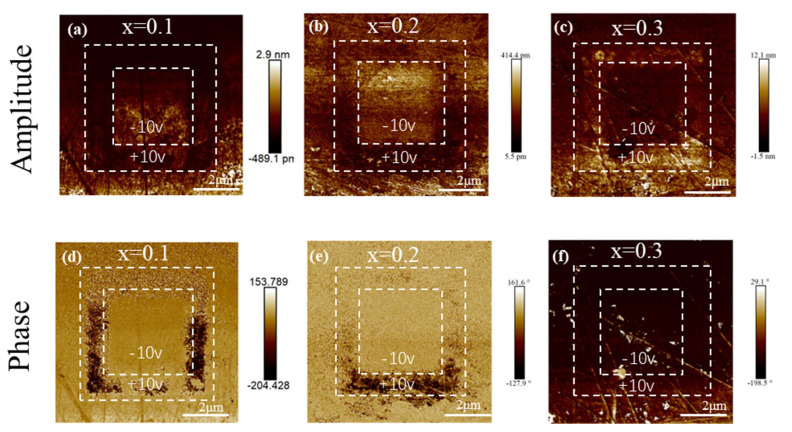
Out-of-plane PFM (**a**–**c**) amplitude and (**d**–**f**) phase images of (1-x)PBSC–xNN ceramics measured under the poling field of ±10 V.

Local piezo-responses (PR) and domain structure information of the PBSC–NN ceramics can be learned from the PFM amplitude, phase images (Figure 8), and the piezo-response loops (Figure S3). It is found that, at x = 0.1, there is clear ferroelectric switching under a changing field of ± 10 V (Figure 8d), and the corresponding PR amplitude is strong (Figure S3a). However, such a feature seemingly weakens with increasing NN content (Figure 8e,f), and a decrease of strain is also observed (Figure S3b,c). This is caused by the reduced ferroelectric domain size as the ferroelectric macrodomains are fragmented into nano-sized PNRs with increasing NN, which are too small to be resolved by PFM. The reduction in domain size combined with diminished phase contrast and PR amplitude have also been reported in many other systems [36,52,53]. At x = 0.3, the relaxor ferroelectricity is further enhanced, and thus, no visible domains can be observed (Figure 8f), but its ferroelectricity nature can be confirmed by the macroscopic P-E measurement result (Figure 5c) and visible PR amplitude contrast (Figure 8c) and loop (Figure S3c,f).

## 4. Conclusions

In conclusion, the present work investigates the energy storage properties of a new ferroelectric crossover PBSC–NN, combining both high entropy relaxor-like ferroelectric (Pb_0.25_Ba_0.25_Sr_0.25_Ca_0.25_)TiO_3_ and the antiferroelectric NaNbO_3_. XRD and Raman spectra confirm that there exists a macroscopical cubic matrix and local tetragonal/pseudo-cubic phases. Moreover, apparent frequency dispersion and characteristic near-zero-field current peak are observed, clearly suggesting relaxor ferroelectricity in this material. It is found that, as NN increases, the entropy value rises. The 0.8PBSC–0.2NN ceramic attains the highest energy storage efficiency (91%), and the 0.7PBSC–0.3NN ceramic with the highest configurational entropy (*S_config_* = 2.19R) realizes the maximum recoverable energy density (2.4 J/cm^3^). This demonstrates that the increase in the entropy value indeed enhances the energy storage performance of the material. Therefore, this study provides a useful reference for developing high-performance electrostatic ceramic capacitors by incorporating a high entropy effect and REF–AFE crossover designs.

## Data Availability

The original contributions presented in this study are included in the article/Supplementary Material. Further inquiries can be directed to the corresponding authors.

## Supplementary Materials

The following supporting information can be downloaded at: https://www.mdpi.com/article/10.3390/ma18091937/s1, Figure S1. Rietveld refinement of the XRD patterns of (1-x)PBSC–xNN ceramics at (a) x = 0.1, (b) x = 0.2 and (c) x = 0.3. Figure S2. Grain size distribution of (1-x)PBSC–xNN ceramics at (a) x = 0.1, (b) x = 0.2 and (c) x = 0.3. Figure S3. Piezo-response (a–c) amplitude and (d–f) phase loops of (1-x)PBSC–xNN ceramics.
