# Supplementary material for: Enhanced Energy Storage Properties of the Relaxor and Antiferroelectric Crossover Ceramic Enabled by a High Entropy Design
Source: Materials (Basel). 2025 Apr 24;18(9):1937. doi: 10.3390/ma18091937 (PMC12072262; doi:10.3390/ma18091937)
Supplement: Supplementary file 1 [file materials-18-01937-s001.zip › materials-3538505-supplementary.pdf]

materials

Supplementary information

Enhanced Energy Storage Properties of the Relaxor and Antiferroelectric Crossover Ceramic Enabled by a High Entropy Design

Yinghao Li [1,2], Wei Xiong [2,3,*], Xuefan Zhou [1], Hang Luo [1], Ru Guo [4,*] and Dou Zhang [1]

1 Powder Metallurgy Research Institute, State Key Laboratory of Powder Metallurgy, Central South University, Changsha 410083, China; yinghaoli@csu.edu.cn (Y.L.); zhouxuefan@csu.edu.cn (X.Z.); hangluo@csu.edu.cn (H.L.); dzhang@csu.edu.cn (D.Z.)
2 Light Alloy Research Institute, Central South University, Changsha 410083, China
3 State Key Laboratory of Precision Manufacturing for Extreme Service Performance, Central South University, Changsha 410083, China
4 Department of Mechanical and Automation Engineering, The Chinese University of Hong Kong, Shatin, Hong Kong, China
* Correspondence: wxiong@csu.edu.cn (W.X.); ruguo@cuhk.edu.hk (R.G.)

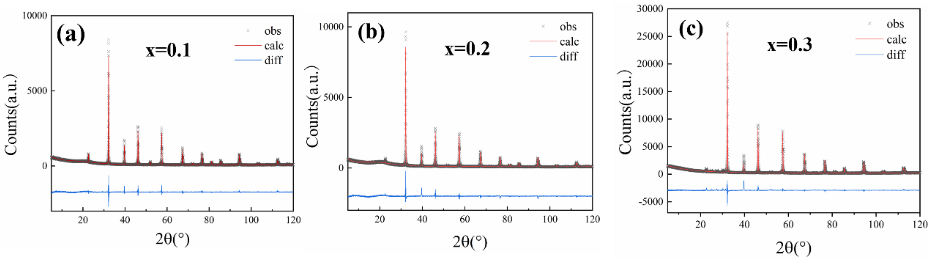

Figure S1. Rietveld refinement of the XRD patterns of (1-x)PBSC-xNN ceramics at (a) x=0.1, (b) x=0.2 and (c) x= 0.3.

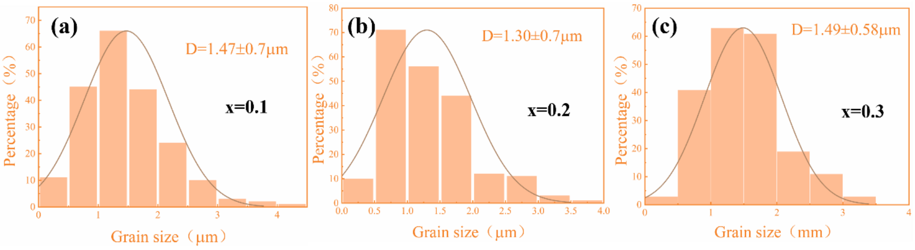

Figure S2. Grain size distribution of (1-x)PBSC-xNN ceramics at (a) x=0.1, (b) x=0.2 and (c) x=0.3.

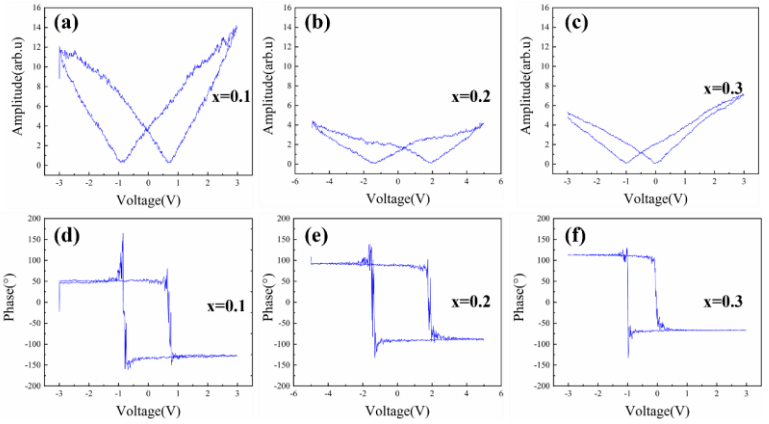

Figure S3. Piezo-response (a, b, c) amplitude and (d, e, f) phase loops of (1-x)PBSC-xNN ceramics.

 https://doi.org/10.3390/ma18091937
